# LIMD1‐AS1 suppressed non‐small cell lung cancer progression through stabilizing LIMD1 mRNA via hnRNP U

**DOI:** 10.1002/cam4.2898

**Published:** 2020-04-02

**Authors:** Jianyuan Pan, Yongqin Tang, Shumei Liu, Lily Li, Bo Yu, Yuanyuan Lu, Yu Wang

**Affiliations:** ^1^ Cardiovascular Department The Second People’s Hospital of Hefei Hefei China; ^2^ General Surgery The First People’s Hospital of Chuzhou Chuzhou China; ^3^ Respiratory Medicine Department Liaocheng People's Hospital Liaocheng China

**Keywords:** hnRNP U, LIMD1, LIMD1‐AS1, NSCLC

## Abstract

**Background:**

Non‐small cell lung cancer (NSCLC) occupies the majority of lung cancer cases and is notorious for the awful prognosis. LIM domains‐containing 1 (LIMD1) is suggested as a tumor suppressor in lung cancer, but its mechanism in NSCLC remains elusive. Present study aimed to uncover the mechanism of LIMD1 in NSCLC.

**Methods:**

qRT‐PCR was performed to analyze the level of LIMD1. The functions of LIMD1 in NSCLC cells were evaluated by CCK‐8, EdU, and caspase‐3 activity assays. RIP and pull‐down assays were applied to determine the interaction of LIMD1 with heterogeneous nuclear ribonucleoprotein U (hnRNP U) and LIMD1‐AS1.

**Results:**

LIMD1 was downregulated in NSCLC samples and cells. Functionally, LIMD1 hindered proliferation and drove apoptosis in NSCLC cells. Moreover, long noncoding RNA (lncRNA) LIMD1 antisense RNA 1 (LIMD1‐AS1) was downregulated in NSCLC samples and cell lines. LIMD1‐AS1 knockdown abrogated NSCLC cell growth in vitro and in vivo. Mechanistically, LIMD1‐AS1 stabilized LIMD1 mRNA through interacting with hnRNP U. Rescue experiments suggested that LIMD1‐AS1 repressed NSCLC progression through LIMD1.

**Conclusions:**

LIMD1‐AS1 suppressed NSCLC progression through stabilizing LIMD1 mRNA via hnRNP U, providing new thoughts for the improvement of molecular‐targeted therapy for NSCLC.

## Graphical abstract

LIMD1 was downregulated in NSCLC, inhibited proliferation, and facilitated apoptosis of NSCLC cells. LIMD1‐AS1 was downregulated in NSCLC and positively regulated LIMD1 expression. LIMD1‐AS1 stabilized LIMD1 mRNA through interacting with hnRNP U.

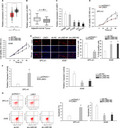

## 1 INTRODUCTION

Lung cancer (LC) is recognized as the commonest cancer type and the predominant cause of tumor‐related mortality.^1^ Statistics show that about 80% of LC cases are NSCLC.^2^ Although recent years have seen the encouraging advances in diagnosis and treatment tools, patients with NSCLC are still faced with unsatisfactory long‐term prognosis.^3^ Hence, the molecular mechanism research on NSCLC is required to improve the prognosis of NSCLC.

LIM domains‐containing 1 (LIMD1) belongs to Ajuba family of LIM domain‐containing proteins.^4^, ^5^ A number of studies reveal that deletion of LIMD1 results in aberrant cellular behaviors, such as cell cycle perturbation and hyperproliferation of cells.^6^ The tumor‐suppressive impact of LIMD1 is supported by studies in multiple cancers, such as gastric cancer and cervical cancer.^7^, ^8^ In NSCLC, evidence proves that LIMD1 is downregulated and results in the misregulation of retinoblastoma protein (pRB) and cell cycle.^9^, ^10^ Moreover, overexpression of LIMD1 in A549 lung cancer cell line hinders tumor growth both in vitro and in vivo.^9^ However, the how LIMD1 expression is modulated in NSCLC remains elusive.

Long noncoding RNAs (lncRNAs) are the transcripts incapable of translating proteins and are 200 bases long.^11^, ^12^ As documented in numerous studies, they are vital modulators of cancer‐related biological activities, including apoptosis, cell growth, invasion, and migration.^13^, ^14^, ^15^ Also, lncRNAs implicated in NSCLC are reported accumulatively. For example, silencing lncRNA PVT1 inhibits NSCLC development by regulating miR‐497.^16^ SNHG1 induces NSCLC progression through blocking miR‐101‐3p and activating Wnt/β‐catenin pathway.^17^ LIMD1‐AS1 is first discovered by our study as an lncRNA nearby LIMD1, and its role in NSCLC is never explained. We are interested in LIMD1‐AS1 because antisense RNAs are reported to modulate their nearby genes,^18^ which indicates that LIMD1‐AS1 potentially modulates LIMD1 in NSCLC.

Heterogeneous nuclear ribonucleoprotein U (hnRNP U), also known as scaffold attachment factor (SAF)‐A, participates in the regulation of mRNA transporting and processing.^19^, ^20^, ^21^ Recent reports show the implication of hnRNP U in modulating DNA damage response and cell apoptosis.^22^, ^23^ In lung cancer, hnRNP U is reported to be downregulated,^24^ and reduces β‐catenin expression by β‐TrCP to inhibit lung cancer progression.^25^ Notably, hnRNP U is a regulator of mRNA stability.^24^, ^26^, ^27^ However, the interaction of hnRNP U with LIMD1 and LIMD1‐AS1 has not been reported.

The goal of this paper is to demonstrate the influences of LIMD1‐AS1 on NSCLC development and to clarify the regulation of LIMD1‐AS1 on LIMD1. Mechanistically, RIP assay measured the interaction between LIMD1 mRNA and hnRNP U. Pull‐down assay evaluated the interaction between LIMD1‐AS1 and LIMD1 mRNA. Function of LIMD1‐AS1 was investigated in vitro by CCK‐8, EdU, and caspase‐3 assays and explored in vivo by establishing xenografts in mice.

## 2 MATERIALS AND METHODS

### 2.1 Bioinformatics analysis

The downregulation of LIMD1 and LIMD1‐AS1 in NSCLC, along with their positive correlation were predicted by GEPIA (http://gepia.cancer-pku.cn/index.html). LIMD1‐AS1 was found to be the neighbor gene of LIMD1 via UCSC tool (http://genome.ucsc.edu/). In addition, Starbase (http://starbase.sysu.edu.cn/) predicted that hnRNP U was a (RNA binding protein) RBP of LIMD1.

### 2.2 Tissue collection

The NSCLC specimens and matched adjacent normal specimens were dissected from 46 NSCLC patients in the Second People's Hospital of Hefei, and they all received no other radiotherapy or chemotherapy before. Signed consents were obtained from all patients and experiment procedure was permitted by ethic committee of the Second People's Hospital of Hefei. The specimens collected were immediately stored under −80℃ in nitrogen until experiments were unfolded.

### 2.3 Cell culture

Human bronchial epithelial cell (16HBE), human NSCLC cells (A549, H1299, H23, and SPC‐A1), and human renal epithelial cell (293T) were all attained from the Shanghai Cell Bank of the Chinese Academy of Sciences (Shanghai, China). 293T and 16HBE cells were cultured in Dulbecco's modified Eagle's medium (DMEM; HyClone) with 10% fetal bovine serum (FBS; Thermo Fisher Scientific) plus 1% penicillin–streptomycin antibiotics (HyClone). A549, H1299, H23, and SPC‐A1 cells were all grown in RPMI 1640 medium (Invitrogen) containing 10% FBS and 1% antibiotics. All media mentioned above were changed every 3 days. Cells were incubated under a humidified air with 5% CO_2_ at 37°C.

### 2.4 Cell transfection

SPC‐A1 or A549 cells were put into 6‐well plates when cell confluence reached 50%‐80%. To increase the expression of LIMD1, LIMD1‐AS1, or hnRNP U, the pcDNA3.1/LMID1, pcDNA3.1/LIMD1‐AS1, and pcDNA3.1/hnRNP U vectors (Genechem) and their corresponding empty vectors were transfected into SPC‐A1 or A549 cells. The screen of stable cells transfected with pcDNA3.1/LMID1, pcDNA3.1/LIMD1‐AS1, and pcDNA3.1/hnRNP U was conducted using G418 (Invitrogen Life Technologies). The short hairpin RNAs (shRNAs) against LIMD1 (sh‐LIMD1#1: CCGGGATCTACTGTGTCCGAGATTACTCGAGTAATCTCGGACACAGTAGATCTTTTTG, sh‐LIMD1#2: CCGGGCCTCACTCATGGAGACTATTCTCGAGAATAGTCTCCATGAGTGAGGCTTTTTG), LIMD1‐AS1 (sh‐LIMD1‐AS1#1: GGTTGTACGGAAATGGGTTCC, sh‐LIMD1‐AS1#2: GCTAACCAACACTGGCTATTA) or HNRNPU (sh‐hnRNP U #1: CCGGCAGTGCTTCTTCCCTTACAATCTCGAGATTGTAAGGGAAGAAGCACTGTTTTTG, sh‐hnRNP U #2: CCGGGCAACTGTGAGACTGAAGATTCTCGAGAATCTTCAGTCTCACAGTTGCTTTTTG), along with controls (sh‐NC), were constructed simultaneously by Genechem to silence LIMD1 or LIMD1‐AS1 expression. Cell transfection was performed in line with the specification of Lipofectamine 2000 Reagent (Invitrogen). Transfection lasted 48 hours.

### 2.5 Quantitative real‐time PCR

SPC‐A1 or A549 cells were subjected to total RNA extraction via a commercially available TRIzol reagent (Invitrogen). First‐strand cDNA was synthesized via reverse transcriptase kit (Takara). To examine the fluorescence intensity of the amplified products, the qRT‐PCR was accordingly conducted on a Step One Applied Biosystems (Applied Biosystems) applying SYBR Green Master (Roche). The reactions were performed as below: at 55°C for 2 minutes, at 95°C for 10 minutes, then 40 cycles of amplification at 95°C for 15 seconds, and ended with 60°C for 1 minute. Relative gene expression was analyzed by the 2^−ΔΔCt^ method, while housekeeping gene GAPDH served as the loading control. Primers are:

**Table:** 

LIMD1	5′‐TGGGGAACCTCTACCATGAC‐3′ (forward)
	5′‐CACAAAACACTTTGCCGTTG‐3′ (reverse)
LIMD1‐AS1	5′‐TAGGGGTGAGGGGTAAGTGG‐3′ (forward)
	5′‐CTTCATGCCAGAAACTGCTCT‐3′ (reverse)
hnRNP U	5′‐GAGCATCCTATGGTGTGTCAAA‐3′ (forward)
	5′‐TGACCAGCCAATACGAACTTC‐3′ (reverse)
GAPDH	5′‐GGGAGCCAAAAGGGTCAT‐3′ (reverse)
	5′‐GAGTCCTTCCACGATACCAA‐3′ (reverse)

### 2.6 Cell viability assay

SPC‐A1 or A549 cells were reaped after transfection and seeded into 96‐well plates when cell density reached 2000 cells per well. Cell Counting Kit‐8 solution (CCK‐8; Dojindo) without FBS was added to each well after 24, 48, 72, and 96 hours. Absorbance was examined at 450 nm wavelength via a microplate reader (Bio‐Tek Instruments).

### 2.7 Caspase‐3 activity assay

SPC‐A1 or A549 cells were added with 1 mL phosphate buffer saline (PBS; Thermo Fisher Scientific). Cells were centrifuged for 10 minutes at 4°C after homogenization. Pellets were cultivated in lysis buffer (Beyotime). Caspase‐3 activity assay was carried out in 96‐well plates with lysate, assay buffer, and Caspase‐3 colorimetric substrate Ac‐DEVD‐pNA at 37°C for 2 hours. The chromospheres pNA was cleaved from the substrate molecule. The absorbance values were measured at a wavelength of 405 nm.

### 2.8 Flow cytometry apoptosis assay

After 48 hours of indicated transfections, collected cells were subjected to a FITC‐Annexin V Apoptosis Detection Kit (BD Biosciences) for analysis following manufacturer's recommendation. For double staining of cells, FITC‐Annexin V as well as propidium iodide (PI) was used. Then, the apoptosis was analyzed by flow cytometry (FACScan; BD Biosciences) and the CellQuest software (BD Biosciences). Cells that were viable, early/late apoptotic, or dead were divided, and apoptosis ratio was considered as the percentage of early/late apoptotic ones.

### 2.9 EdU staining

EdU staining was carried out to evaluate the proliferative capacity. The Click‐iT Alexa Fluor 488 Imaging Kit (Invitrogen) was utilized in accordance with the standard method. Transfected SPC‐A1 or A549 cells were initially rinsed with 3% bovine serum albumin (BSA; Sigma‐Aldrich, Burlington, Massachusett, USA) twice and then treated with 150 μL EdU‐Click reaction‐mix at 20°C for 30 minutes, followed by washing with 3% BSA. Cell nuclei were treated with DAPI staining in the dark.

### 2.10 Animal xenografts

Twelve 4‐week‐old athymic BALB/c nude mice (female) obtained from Shanghai Laboratory Animal Center were kept in three disposable plastic cages (n = 4 mice per cage) under pathogen‐free conditions with Sani‐Chip bedding. Before being utilized, the mice were housed in cages for 7 days to accommodate the environment. SPC‐A1 cells were stably transfected with pcDNA3.1/LIMD1‐AS1 (experimental group, n = 6) and pcDNA3.1 empty vector (control group, n = 6), and collected from 6‐well cell culture plates. After that, cells were washed using phosphate‐buffered saline, followed by cell resuspension. Stably expressed cells after transfection were subcutaneously injected into both sides of each mouse. Every 3 days, tumor growth (primary outcome assessed) was monitored and the equation V = 0.5 × D × d^2^ (V, volume; d, latitudinal diameter D; longitudinal diameter) was used for tumor volume calculation (secondary outcome assessed). Twenty‐eight days’ postinjection, isoflurane anesthesia overdose inhalation was applied to euthanized mice, and subcutaneous tumor growth obtained from euthanized mice underwent further examination. Following the Guide for the Care and Use of Laboratory Animals of the National Institutes of Health, this study was conducted. The researchers who monitored and recorded cell growth were unaware of the accommodation and injection condition of mice.

### 2.11 Immunohistochemical staining

To test Ki‐67 level, tissue samples were stained. Primary monoclonal probes for Ki‐67 were used for tumor detection at 4°C overnight. After that, a suitable second antibody was used for incubation. Tissues underwent diaminobenzidine treatment and hematoxylin counterstaining. A microscope (Carl Zeiss) was applied for tissue observation.

### 2.12 Luciferase reporter assay

LIMD1 promoter was cloned into the pGL3‐basic to produce pGL3‐LIMD1 reporter. 293T cells were co‐transfected with the pGL3‐LIMD1 vector and pcDNA3.1/LIMD1‐AS1 or sh‐LIMD1‐AS1#1/2, together with their respective controls. Transfection was carried out using Lipofectamine 2000 as per the supplier's recommendation. The Dual Luciferase Reporter Assay System (Promega) was applied after 48 hours transfection as the manufacturer's directions requested.

### 2.13 RIP assay

According to the protocol of EZ‐Magna RIP kit (Millipore), RIP assay was carried out in SPC‐A1 and A549 cells. Cell lysates in RIP buffer were treated with magnetic beads conjugated to antibodies against hnRNP U, IgG as negative control or SNRNP70 as positive control. After treatment with proteinase K (Absin), qRT‐PCR was performed.

### 2.14 Western blotting analysis

To extract protein, RIPA lysis buffer (Solarbio) was applied. Protein was subsequently quantified via BCA protein assay (Beyotime). The separation of protein was performed on 15% SDS‐PAGE gels. Protein was shifted to PVDF membranes (Millipore) afterwards. Then, the membranes were blocked by 5% non‐fat milk in TBST at room temperature for 1 hour and treated with the primary antibodies, namely, anti‐LIMD1 (1/1500, ab155788, Abcam), anti‐hnRNP U (1‐5 μg/mL, SAB1402227, Sigma‐Aldrich) and anti‐GAPDH (1/1000, ab8245, Abcam) as an endogenous control. Before incubating with secondary antibodies, the membranes were rinsed with TBST for three times. The ECL system (Santa Cruz Biotechnology) was utilized to monitor the final protein bands.

### 2.15 RNA pull‐down assay

Plasmids containing LIMD1‐AS1 and antisense LIMD1‐AS1 sequences were linearly cut, transcribed and biotinylated in vitro for 48 hours using a MAXIscript T7 Transcription Kit (Thermo Fisher Scientific). RNA pull‐down assay was conducted using Pierce Magnetic RNA‐Protein Pull‐Down Kit (Thermo Fisher Scientific) in A549 cell lysates. The streptavidin‐coated magnetic beads (Ambion) were added in the cell lysates, following digestion with proteinase K. The pull‐down proteins were subjected to western blot and qRT‐PCR.

### 2.16 Actinomycin D (Act D) treatment

After transfection, A549 cells were collected and planted in the 6‐well plates. 20 μg/mL Act D was incubated with cells to block mRNA transcription. At the indicated time points, cells were harvested. Total RNA was isolated to detect the mRNA stability.

### 2.17 Statistical analysis

Statistical analysis was carried out by SPSS 23.0 (SPSS Inc). Each experiment was conducted for at least three times in this research. The Student's *t* test or one‐way analysis of variance (ANOVA) was used for differences analysis. A value of *P* < .05 was regarded to manifest a statistically significant difference. Results in this research were shown as mean ± standard deviation (SD).

## 3 RESULTS

### 3.1 LIMD1 was low expressed in NSCLC, and its overexpression hindered proliferation and drove apoptosis of NSCLC cells

**Figure 1:**
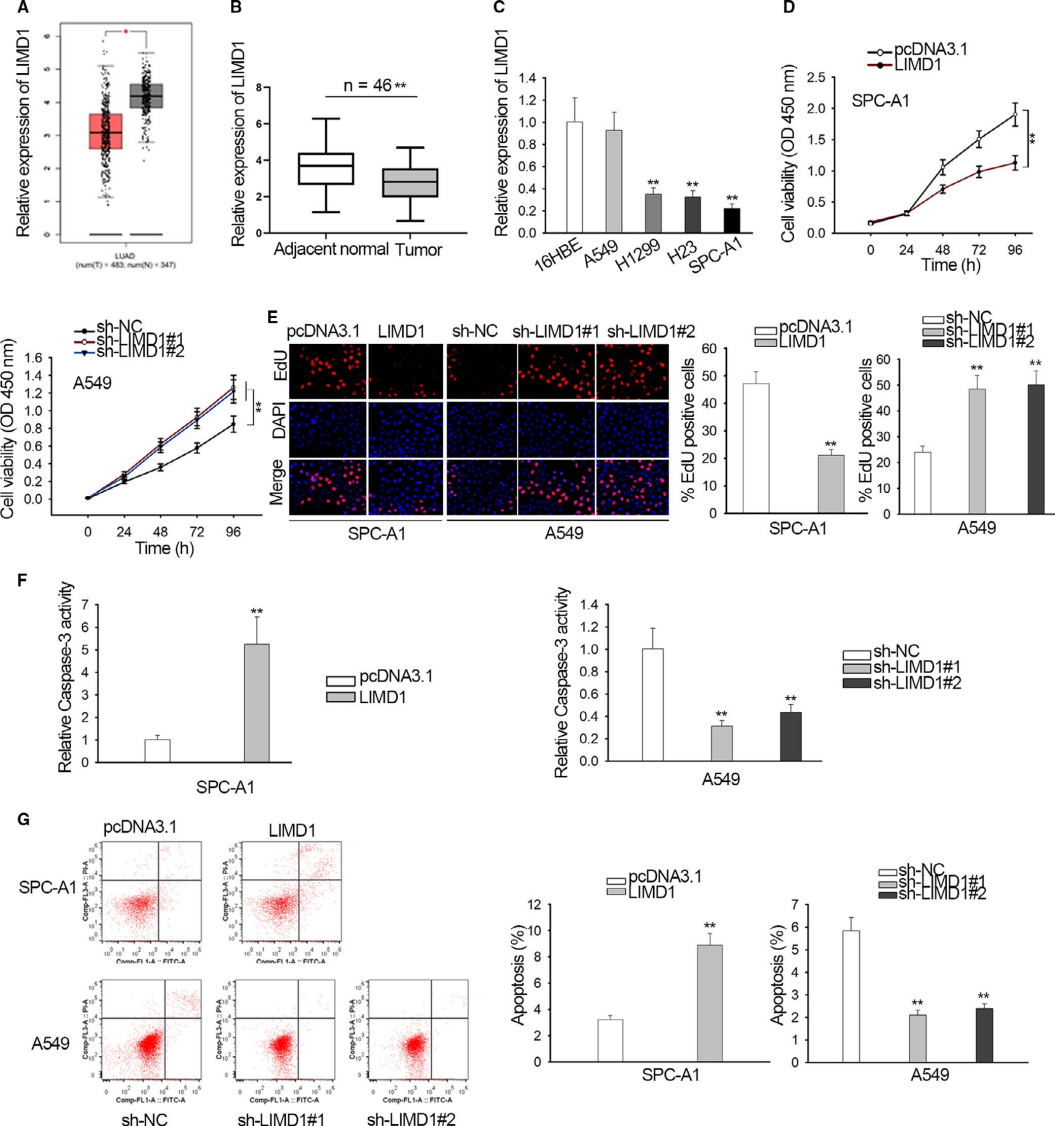
LIMD1 expression and function in non‐small cell lung cancer (NSCLC). A, LIMD1 level in lung adenocarcinoma lung adenocarcinoma samples was analyzed based on TCGA data in online bioinformatics tool GEPIA. B, LIMD1 level in NSCLC specimens versus para‐tumor specimens was analyzed by qRT‐PCR. (C) qRT‐PCR data of LIMD1 level in NSCLC cell lines versus normal cell line. D‐E, Viable cells was determined by CCK‐8 and EdU‐labeled proliferative cells were pictured and quantified in SPC‐A1 and A549 cells upon overexpression and knockdown of LIMD1. F, Caspase‐3 activity was analyzed in SPC‐A1 and A549 cells upon LIMD1 overexpression and knockdown. G, Flow cytometry plot and quantification of apoptotic SPC‐A1 and A549 cells upon LIMD1 overexpression and knockdown. *P < .05, **P < .01

To establish association between LIMD1 and NSCLC, we determined LIMD1 expression. By analyzing TCGA data using GEPIA (http://gepia.cancer-pku.cn/), we obtained the downregulation of LIMD1 in lung adenocarcinoma (LUAD) samples compared with the normal samples (Figure 1A). Consistently, LIMD1 was also downregulated in specimens form NSCLC patients (Figure 1B). Besides, we observed the low LIMD1 level in NSCLC cell lines (A549, H1299, H23, and SPC‐AS1) versus the normal 16HBE cells (Figure 1C). Thus, we indicated that LIMD1 participated in NSCLC.

Next, impact of LIMD1 in NSCLC was tested in vitro. We overexpressed LIMD1 in SPC‐A1 which exhibited the lowest LIMD1 level, and silenced LIMD1 in A549 which exhibited the highest LIMD1 level. qRT‐PCR analysis confirmed the elevation of LIMD1 level SCP‐A1 cells under pcDNA3.1/LIMD1 transfection and the decline of LIMD1 level in A549 cells under sh‐LIMD1#1/2/3 transfection, and sh‐LIMD1#1/2 exhibited better knockdown efficiency (Figure S1A). The EdU‐labeled SPC‐A1 cells and colonies from SPC‐A1 cells were decreased upon LIMD1 overexpression, and opposite result were observed in A549 cells with LIMD1 silence (Figure 1D,E). The caspase‐3 activity in SPC‐A1 cells increased responding to LIMD1 overexpression, whereas that in A549 cells decreased under LIMD1 silence (Figure 1F). Additionally, apoptotic SPC‐A1 cells presented an increased ratio under LIMD1 overexpression, and apoptotic A549 cells exhibited a decreased ratio under LIMD1 silence (Figure 1G). Therefore, LIMD1 functioned as a suppressor in NSCLC by abrogating proliferation and strengthening apoptosis.

### 3.2 LIMD1‐AS1 was downregulated in NSCLC and positively regulated LIMD1 by stabilizing its mRNA

**Figure 2:**
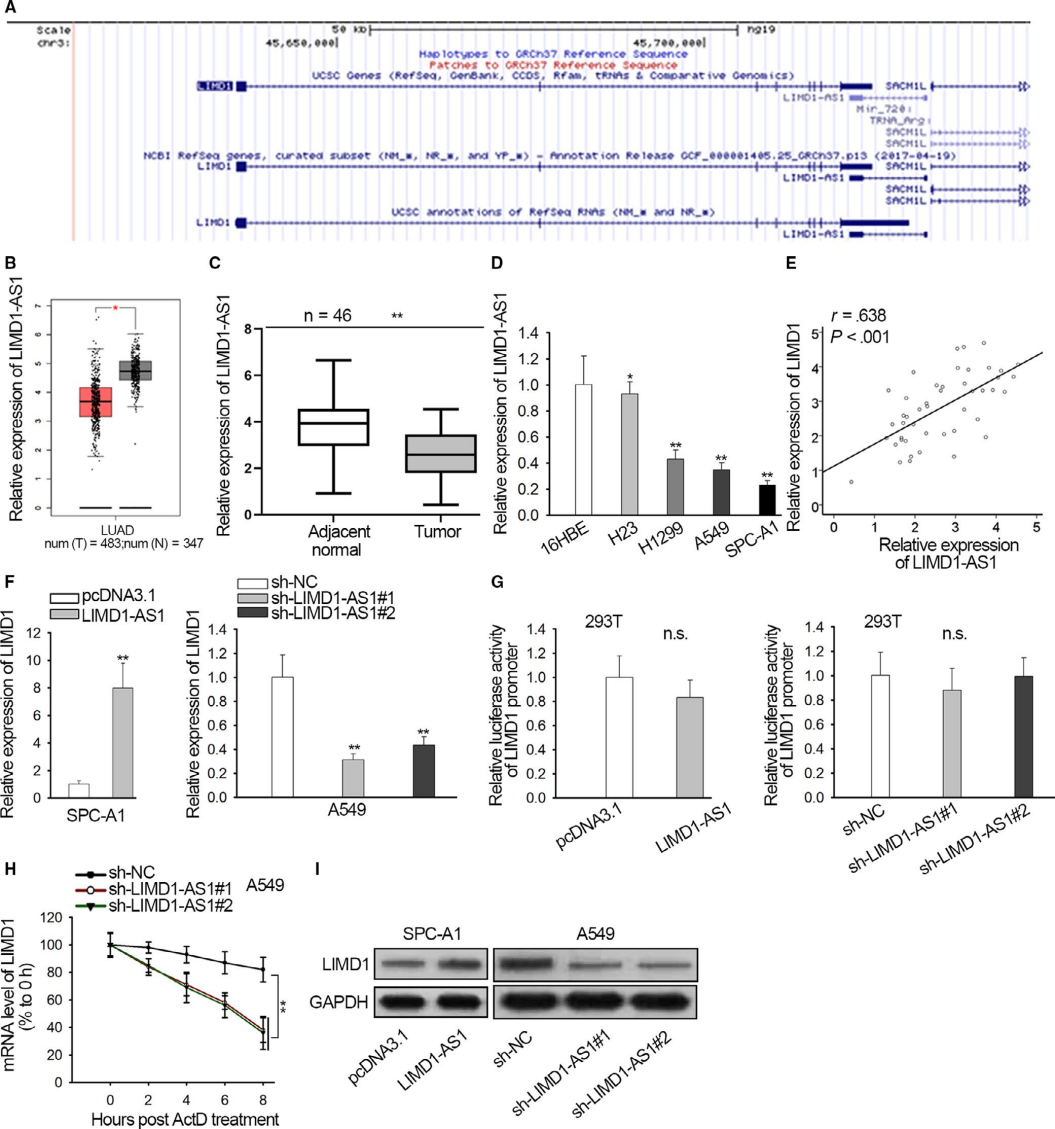
LIMD1‐AS1 was downregulated in non‐small cell lung cancer (NSCLC) and positively regulated LIMD1 by stabilizing its mRNA. A, UCSC data showed the neighboring genomic location of LIMD1‐AS1 and LIMD1. B, LIMD1‐AS1 level in lung adenocarcinoma samples from TCGA data was analyzed by online bioinformatics tool GEPIA. C, qRT‐PCR data of LIMD1‐AS1 level in NSCLC specimens versus para‐tumor ones. D, qRT‐PCR data of LIMD1‐AS1 expression in NSCLC cell lines versus normal cell line. E, qRT‐PCR was used to determine the overexpression and knockdown of LIMD1‐AS1 in SPC‐A1 and A549 cells. F, Spearman's correlation curve presented positive LIMD1‐AS1 and LIMD1 expression correlation in NSCLC specimens. G, Luciferase reporter assay was applied to determine the effect of LIMD1‐AS1 on promoter transcription of LIMD1 in 293T cells. H, Act D was added to block mRNA production. The level of remaining LIMD1 mRNA was evaluated by qRT‐PCR every 2 h in A549 cells upon LIMD1‐AS1 silence. I, Protein content of LIMD1 was monitored upon the altered level of LIMD1‐AS1. *P < .05, **P < .01. n.s.: no significance

Thereafter, we investigated the upstream mechanism of LIMD1 in NSCLC. Through browsing UCSC (http://genome.ucsc.edu/), we discovered that the genomic location of LIMD1‐AS1 neighbored LIMD1 (Figure 2A). It has been reported that antisense RNAs could potentially regulate the expression of their neighbor genes.^18^, ^28^ Therefore, we assumed the correlation between LIMD1 and LIMD1‐AS1. First, interrogated the implication of LIMD1‐AS1 in NSCLC. According to TCGA data analysis in GEPIA, LIMD1‐AS1 level was LOW in LUAD samples (Figure 2B). qRT‐PCR data exhibited the suppressed LIMD1‐AS1 level in NSCLC specimens versus para‐tumor ones (Figure 2C). We also validated the downregulation of LIMD1‐AS1 in NSCLC cell lines (Figure 2D). Moreover, we validated positive correlation between LIMD‐AS1 and LIMD1 in LUAD through GEPIA (Figure S1B), and confirmed their positive association in NSCLC tissues (Figure 2E).

Then, the regulation of LIMD1‐AS1 on LIMD1 was investigated. We induced LIMD1‐AS1 level in SPC‐A1 cells and silenced endogenous LIMD1‐AS1 expression in A549 cells (Figure S1C). It turned out that level of LIMD1 mRNA and protein increased under LIMD1‐AS1 overexpression, but declined under LIMD1‐AS1 knockdown (Figure 2F). Regulatory mechanisms of lncRNAs at transcriptional and posttranscriptional levels are extensively reported.^29^ Hence, we further detected at which level LIMD1‐AS1 modulated LIMD1 expression. We discovered that luciferase activity of LIMD1 promoter was not altered under either overexpression or depletion of LIMD1‐AS1 (Figure 2G), indicating that LIMD1‐AS1 regulated LIMD1 posttranscriptionally. Therefore, we tested whether LIMD1‐AS1 affected LIMD1 mRNA stability. After adding Act D to block mRNA synthesis, the remaining LIMD1 mRNA level of was detected by qRT‐PCR. Consequently, the mRNA stability of LIMD1 was abrogated by LIMD1‐AS1 silence (Figure 2H). Furthermore, LIMD1 protein content increased when LIMD1‐AS1 was overexpressed, and declined upon LIMD1‐AS1 depletion (Figure 2I).

Additionally, function of LIMD1‐AS1 was tested in vivo. Results indicated that SPC‐A1 cells with LIMD1‐AS1 overexpression formed smaller tumors in mice than control group (Figure S2A). The volume of tumors derived from LIMD1‐AS1 overexpressing cells (n = 6) were smaller than that derived from control cells (n = 6) (Figure S2B). The tumor weight in LIMD1‐AS1 overexpressing group ($\bar{x}$ = 301, s = 33) was lighter than that in control group ($\bar{x}$ = 622, s = 64) (Figure S2C). Immunohistochemical staining also exhibited the reduced positivity of Ki‐67 in LIMD1‐AS1 overexpression group in comparison to control group (Figure S2D). Collectively, these data implied that LIMD1‐AS1 was downregulated in NSCLC and positively regulated LIMD1 by stabilizing its mRNA. Besides, LIMD1‐AS1 overexpression impeded tumorigenesis in NSCLC.

### 3.3 LIMD1‐AS1 stabilized LIMD1 mRNA by interacting with hnRNP U

**Figure 3:**
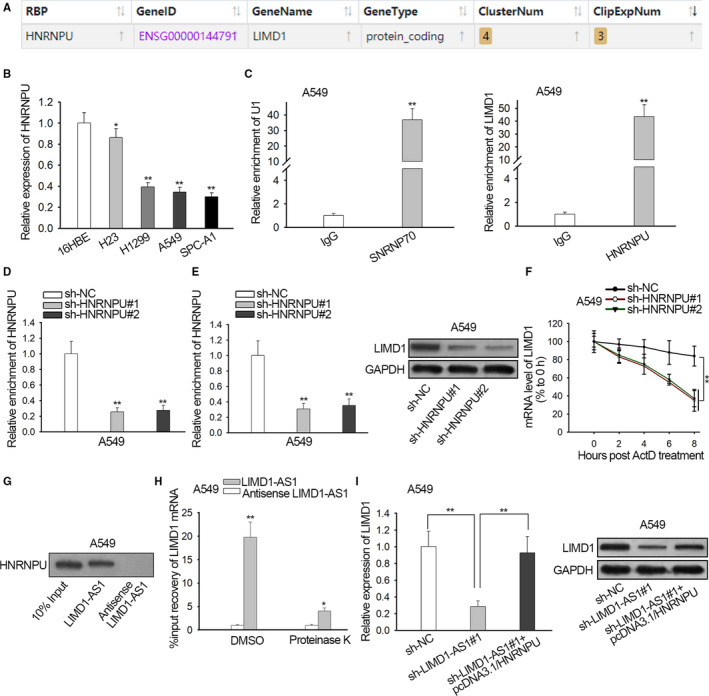
LIMD1‐AS1 stabilized LIMD1 mRNA by interacting with hnRNP U. A, Potential interaction between hnRNP U and LIMD1 mRNA was predicted by starBase 3.0. B, qRT‐PCR analysis of hnRNP U level in non‐small cell lung cancer cell lines versus the normal cell line. C, qRT‐PCR analysis of LIMD1 mRNA enrichment in RIP hnRNP U. SNRNP7 and U1 were positive control. D, The knockdown of hnRNP U was examined by qRT‐PCR analysis in A549 cells. E, qRT‐PCR of LIMD1 mRNA and western blot of LIMD1 protein in A549 cells with hnRNP U silence. F, The level of remaining LIMD1 mRNA was evaluated by qRT‐PCR every 2 h after Act D treatment in A549 cells with hnRNP U silence. G, Western blot of hnRNP U in products pulled down by LIMD1‐AS1 and antisense LIMD1‐AS1. H, qRT‐PCR data of LIMD1 mRNA in the pull‐down of LIMD1‐AS1 and antisense LIMD1‐AS1 group in A549 cells treated with Proteinase K (to digest proteins) or with DSMO control. I, qRT‐PCR and western blot of LIMD1 level in A549 cells transfected with sh‐NC, sh‐LIMD1‐AS1#1, or sh‐LIMD1‐AS1#1 + pcDNA3.1/hnRNP U, respectively. *P < .05, **P < .01

Furthermore, we asked how LIMD1‐AS1 regulated LIMD1 mRNA stability. Mounting reports support that lncRNAs strengthen or impair the mRNA stability through recruiting certain RNA binding proteins (RBPs), including in NSCLC.^24^ Therefore, we hypothesized that LIMD1‐AS1 modulated LIMD1 mRNA stability in this manner. We searched starBase 3.0 (http://starbase.sysu.edu.cn/) to identify the potential RBPs targeting LIMD1, finding that hnRNP U potentially interacted with LIMD1 (Figure 3A). Previous studies report the function of hnRNP U as an mRNA stabilizer.^24^, ^26^, ^27^ Also, some works prove that hnRNP U exerts suppressive effect on NSCLC progression.^24^, ^25^ Therefore, we speculated that LIMD1‐AS1 regulated LIMD1 mRNA stability through hnRNP U. We confirmed the downregulated hnRNP U level in NSCLC cell lines (Figure 3B). qRT‐PCR verified the abundant expression of LIMD1 mRNA in the RIP precipitates of hnRNP U antibody (Figure 3C). Later, we detected the impact of hnRNP U on LIMD1. We silenced hnRNP U expression in A549 cells and confirmed the knockdown efficiency (Figure 3D). Then, we observed that knockdown of hnRNP U reduced the level of LIMD1 mRNA and protein in A549 cells (Figure 3E). Moreover, the half‐life of LIMD1 mRNA was shortened by hnRNP U silence (Figure 3F). Further, the interaction between hnRNP U and LIMD1‐AS1 was investigated. Pull‐down assay revealed that the protein of hnRNP U was enriched in products pulled down by LIMD1‐AS1 rather than antisense LIMD1‐AS1 (Figure 3G). Also, we confirmed the enrichment of LIMD1 mRNA in the pull‐down of LIMD1‐AS1 rather than antisense LIMD1‐AS1, and that the addition of proteinase K markedly reduced the enrichment (Figure 3H), indicating that LIMD1‐AS1 interacted with LIMD1 in an hnRNP U‐required way. Additionally, the overexpression of hnRNP U restored LIMD1 mRNA and protein levels in LIMD1‐AS1‐silenced A549 cells (Figure 3I). In sum, LIMD1‐AS1 stabilized LIMD1 mRNA by interacting with hnRNP U.

### 3.4 LIMD1‐AS1 impeded proliferation and prompted apoptosis through LIMD1 in NSCLC

**Figure 4:**
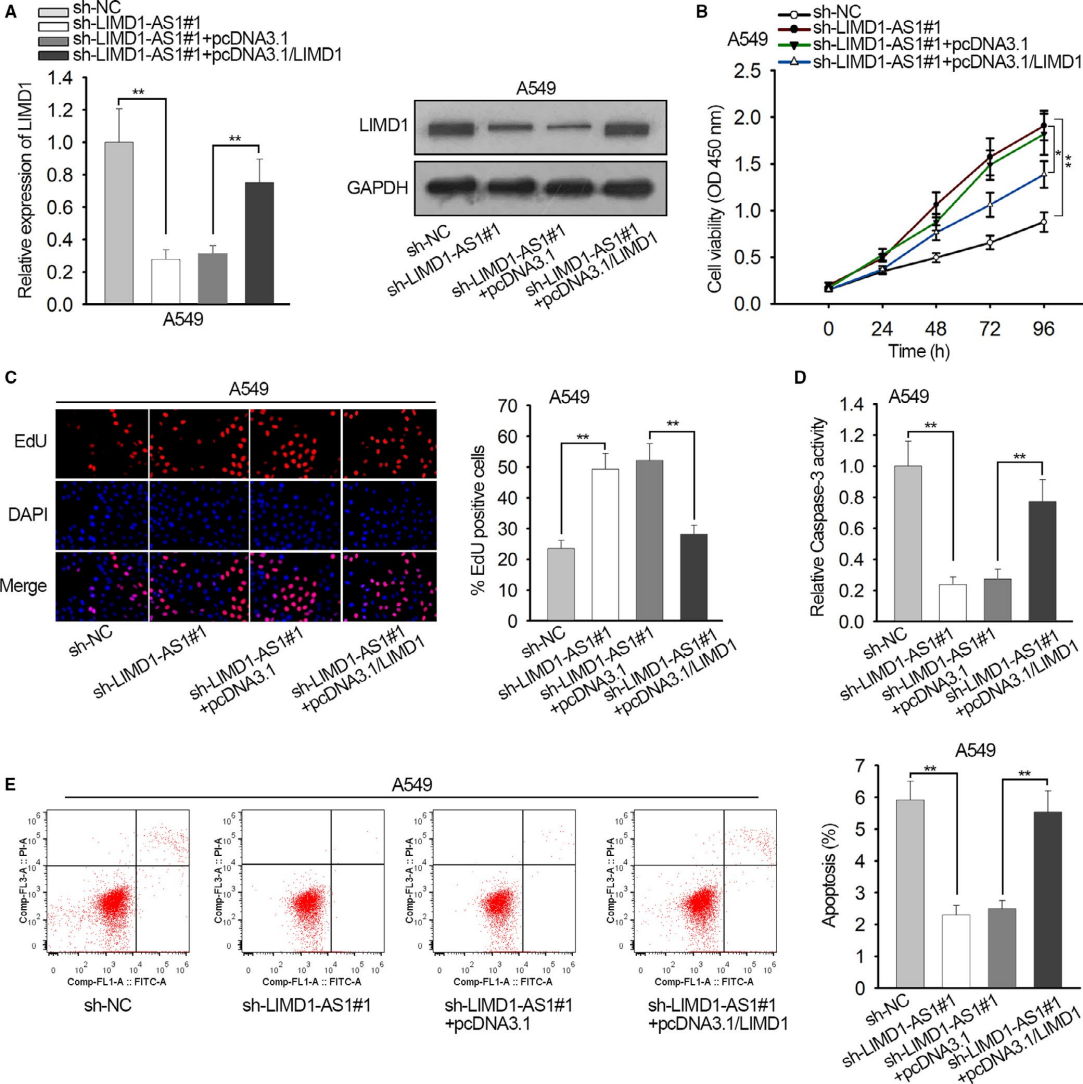
LIMD1‐AS1 impeded proliferation and prompted apoptosis through LIMD1 in non‐small cell lung cancer. A549 cells were transfected with sh‐NC, sh‐LIMD1‐AS1#1, sh‐LIMD1‐AS1#1 + pcDNA3.1, or sh‐LIMD1#1 + pcDNA3.1/LIMD1, respectively. A, qRT‐PCR and western blot data of LIMD1 level in A549 cells of each group. B‐C, CCK‐8 and EdU assays were used to monitor the proliferation of A549 cells of each group. D, Caspase‐3 activity was evaluated in A549 cells of each group. E, Apoptotic A549 cells in each group was analyzed by flow cytometry. *P < .05, **P < .01

Finally, we examined whether LIMD1 mediated the impact of LIMD1‐AS1 on NSCLC progression. We validated that LIMD1 expression reduced by LIMD1‐AS1 was recovered by transfecting pcDNA3.1/LIMD1 into A549 cells (Figure 4A). CCK‐8 and EdU analyses depicted that silencing LIMD1‐AS1 facilitated proliferation of A549 cells, and overexpressing LIMD1 abrogated the facilitating effect of LIMD1‐AS1 knockdown (Figure 4B,C). LIMD1‐AS1 knockdown impaired caspase‐3 activity in A549 cells but the ectopic LIMD1 expression restored caspase‐3 activity versus sh‐LIMD1‐AS1#1 group (Figure 4D). Moreover, overexpressing LIMD1 restored the percentage of apoptotic A549 cells reduced by sh‐LIMD1‐AS1#1 (Figure 4E). Thus, LIMD1‐AS1 impeded proliferation and prompted apoptosis through LIMD1 in NSCLC.

## 4 DISCUSSION

Previous studies have suggested that LIMD1 participates in tumor progression of diverse cancers by serving as a tumor‐suppressor, such as in gastric cancer and cervical cancer.^7^, ^8^ Also, LIMD1 is revealed by former studies to present low expression and hinder tumor growth In NSCLC.^9^, ^10^ These findings indicated that the investigating LIMD1‐related mechanism in NSCLC progression might be helpful to provide new thoughts for molecular‐targeted treatments of NSCLC.

In concordance, we confirmed that LIMD1 was downregulated in LUAD samples according to TCGA data analysis and confirmed its low expression in NSCLC specimens and cells according to qRT‐PCR data. Functionally, we illustrated that LIMD1 inhibited proliferation and drove apoptosis in NSCLC cells, which was also consistent with previous reports.

As suggested by numerous studies over the past decades, lncRNAs are catching more and more attentions of researchers for their rising roles in cancer progression.^13^, ^14^, ^15^ A number of lncRNAs have been revealed as promising biological markers in NSCLC, such as lncRNA PVT1 and SNHG1.^16^, ^17^ Our study first discovered lncRNA LIMD1‐AS1 in cancer progression. We speculated the modulatory function of LIMD1‐AS1 on LIMD1 in NSCLC because former findings imply that lncRNAs could regulate their neighbor genes.^18^, ^28^ We found through UCSC that LIMD1‐AS1 was neighbor to LIMD1 according to their genomic location. Through GEPIA, we first uncovered that LIMD1‐AS1 was downregulated and positively correlated with LIMD1 expression in LUAD samples. We then confirmed that LIMD1‐AS1 positively regulated LIMD1 expression in NSCLC cells at posttranscriptional level by stabilizing LIMD1 mRNA. Additionally, we offered the first data that LIMD1‐AS1 overexpression impaired NSLCL tumorigenesis in vivo, suggesting LIMD1‐AS1 as an antitumor lncRNA.

Mechanistically, lncRNAs are proved by former studies to interact with RBPs to regulate gene expression.^24^ hnRNP U, recognized as a regulator of mRNA transporting and processing,^19^, ^20^, ^21^ was previously reported to perform suppressive roles in cancers by regulating DNA damage response and cell apoptosis.^22^, ^23^ Interestingly, a study has shown that lncRNA SFTA1P promoted apoptosis and increased cisplatin chemosensitivity by stabilizing GADD45A through hnRNP U in lung squamous cell carcinoma.^24^ Accordingly, our study confirmed that hnRNP U was downregulated in NSCLC cells. Importantly, we first demonstrated that LIMD1‐AS1 interacted with hnRNP U to stabilize LIMD1 mRNA. Finally, we suggested through rescue assay that LIMD1 was involved in the negative regulation of LIMD1‐AS1 on NSCLC progression.

One major limitation of our work lies in the sex of the mice utilized in the animal assays. The differences between female and male mice may lead to some unknown random errors, which would be improved in further studies. Also, further research is needed to confirm the functions of LIMD1‐AS1 on human NSCLC cells growth.

Taken together, our study first revealed that LIMD1‐AS1 suppressed NSCLC progression through stabilizing LIMD1 mRNA via hnRNP U, indicating LIMD1‐AS1 as a novel tumor‐suppressor gene in NSCLC progression from in vitro and in vivo analyses.

## CONFLICT OF INTEREST

The authors declare that they have no competing interests.

## AUTHORS’ CONTRIBUTIONS

Conception and design: PJ and TY; Experimentation: PJ and TY; Analysis of data: PJ, TY, LS, LL, and YB; Manuscript the article: LY and WY; Revision and Edition the article: PJ, TY; All authors read and approved the final version of the manuscript.

## ETHICAL APPROVAL

Ethical approval was obtained from committee of the Ethics of Animal Experiments of the Second People's Hospital of Hefei. All procedure regarding animal treatments and the whole study were performed in line with the ethical standards of this committee.

## Data Availability

Research data are not shared.
