# Supplementary figures and images for: LIMD1‐AS1 suppressed non‐small cell lung cancer progression through stabilizing LIMD1 mRNA via hnRNP U
Source: Cancer Med. 2020 Apr 2;9(11):3829–39. doi: 10.1002/cam4.2898 (PMC7286462; doi:10.1002/cam4.2898)

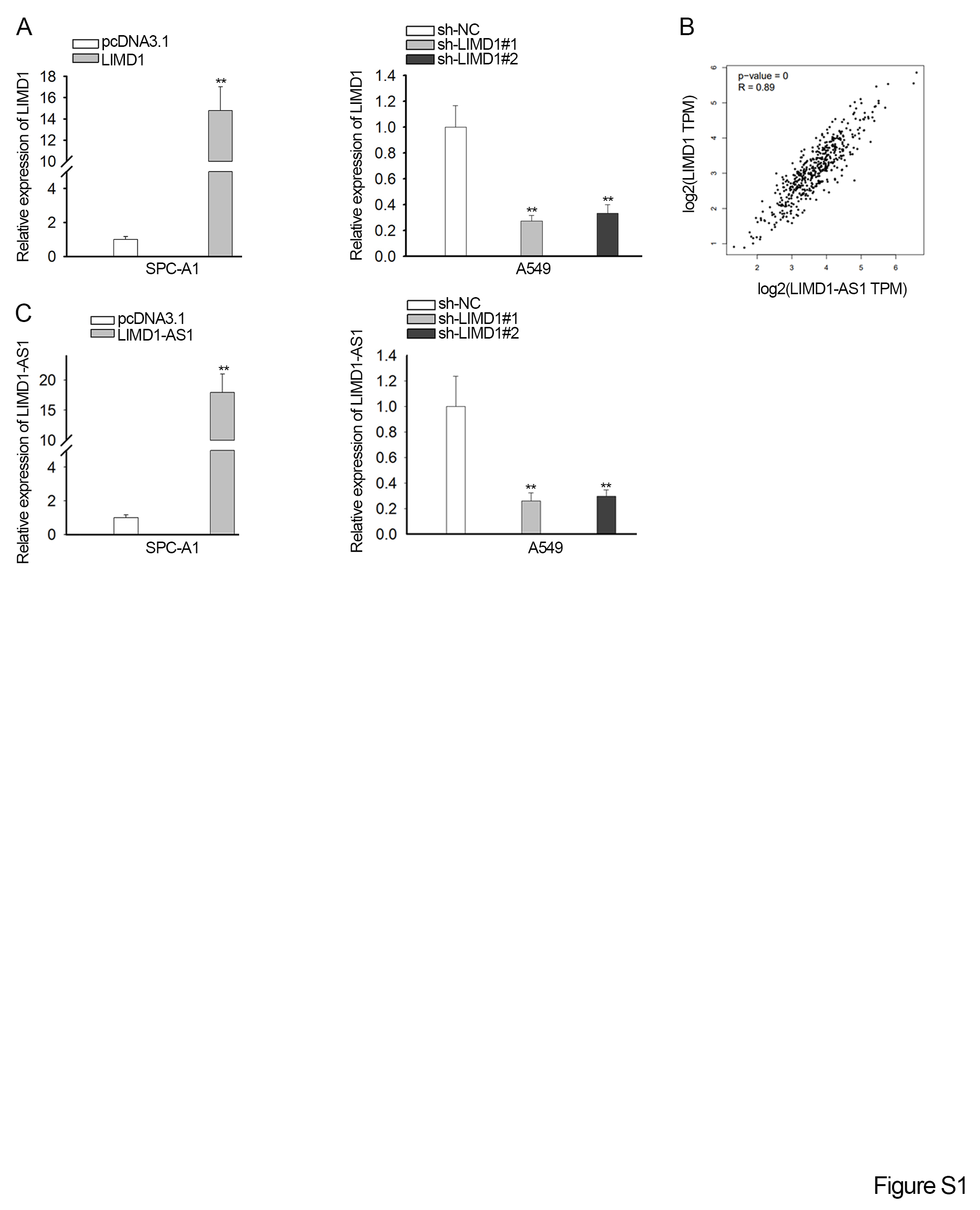

Supplement: Supplementary file 1 [file CAM4-9-3829-s001.tif]

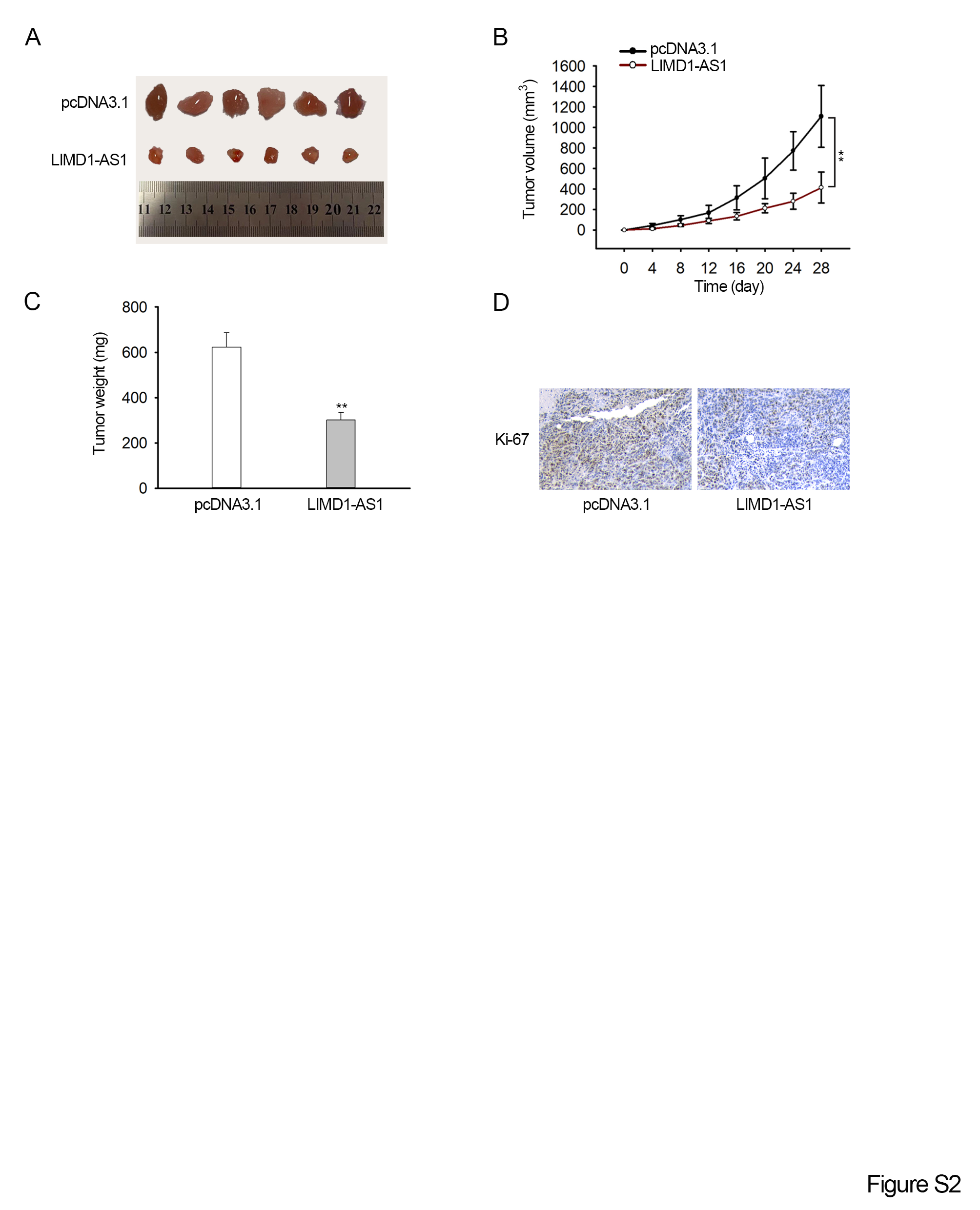

Supplement: Supplementary file 2 [file CAM4-9-3829-s002.tif]
